# Tri-State Medical Society

**Published:** 1898-10

**Authors:** 


### Tri-State Medical Society.

The following are some of the papers which will be read at the Tenth Annual Meeting of the Tri-State Medical Society, which meets in Birmingham, Ala., October 25, 26, and 27.

President's Address. J. A. Goggans, Alexander City, Ala.

"Early Diagnosis of Cancer of the Uterus." Thos. E. Cullen, Baltimore, Md.

"Acute Anterior Poliomyelitis." E. D. Bondurant, Mobile, Ala.

"A Case of Complete Obstruction of the Common Bile Duct by Floating Gall Stone." W. H. Hudson, LaFayette, Ala.

"A Simple Operation for Hemorrhoids Without Injections, Ligature, Clamp, Cautery or Crushing." R. R. Kime, Atlanta, Ga.

"Total Amputation of the Penis so that the Patient can Uurinate Normally." H. M. Hunter, Union Springs, Ala.

"Impotence." W. H. Mangum, Georgiana, Ala.

"Extirpation of the Pancreas." H. Berlin, Chattanooga.

"Two Cases of Surgery." S. W. Purifoy, Lowndesboro, Ala.

"Fracture of the Spine; Presentation of Two Cases. R. G. Copeland, Birmingham, Ala.

"The Treatment of Intestinal Obstruction and Constipation, by Electric Injections." R. P. Johnson, Oak Park, Ill.

"Conservative Gynaecology per Rational Medication." R. H. Hayes, Union Springs, Ala.

"Ectopic Gestation." W. E. B Davis, Birmingham.

"Modern Treatment of Corneal Opacities, with Report of Cases." M. L. Heffelfinger, Huntsville, Ala.

"Keratitis." A. A. Greene, Anniston, Ala.

"Purulent Ophthalmia; New Method of Treatment." Frank Trester Smith, Chatanooga.

"Fevers of Alabama." Charles McAlpine Watson, Florence, Ala.

"Some Fevers of St. Clair County, Alabama." Eugene P. Cason, Ragland, Ala.

"Continued Malarial Fever in Southeast Alabama." Wm. R. Belcher, Daleville, Ala.

"Typhoid Fever." H. Eugene Mitchell, Oneonta, Ala.

"Typhoid Fever." E. A. Matthews, Clanton, Ala.

"Typhoid Fever; Report of Cases." C. L. Guice, Harris, Ala.

"Some Suggestions in the Treatment of Typhoid Fever." J. C. LeGrand, Birmingham.

"Diphtheria." H. L. Appleton, Cedar Bluff, Ala.

"Chorea." S. W. Fain, Chattanooga.

"Suggestion in the Healing Art." E. T. Camp, Gadsden, Ala.

